# Transcript profiling provides insights into molecular processes during shoot elongation in temperature-sensitive peach (*Prunus persica*)

**DOI:** 10.1038/s41598-020-63952-2

**Published:** 2020-05-08

**Authors:** Xiaodong Lian, Bin Tan, Liu Yan, Chao Jiang, Jun Cheng, Xianbo Zheng, Wei Wang, Tanxing Chen, Xia Ye, Jidong Li, Jiancan Feng

**Affiliations:** 1 College of Horticulture, Henan Agricultural University, Zhengzhou, 450002 China; 2 Henan Key Laboratory of Fruit and Cucurbit Biology, Zhengzhou, 450002 China

**Keywords:** Agricultural genetics, Gene expression

## Abstract

Plant growth caused by ambient temperature is thought to be regulated by a complex transcriptional network. A temperature-sensitive peach (*Prunus persica*) was used to explore the mechanisms behind shoot internode elongation at elevated temperatures. There was a significantly positive correlation between the length of the terminal internode (TIL) and the maximum temperature three days prior to the measuring day. Four critical growth stages (initial period and initial elongation period at lower temperature, rapid growth period and stable growth period at higher temperature) were selected for comparative RNA-seq analysis. About 6.64G clean bases were obtained for each library, and 88.27% of the data were mapped to the reference genome. Differentially expressed gene (DEG) analysis among the three pairwise comparisons resulted in the detection of several genes related to the shoot elongation in temperature-sensitive peach. HSFAs were up-regulated in response to the elevated temperature, while the up-regulated expression of HSPs might influence hormone signaling pathways. Most of DEGs involved in auxin, abscisic acid and jasmonic acid were up-regulated, while some involved in cytokinin and brassinosteroid were down-regulated. Genes related to ethylene, salicylic acid and circadian rhythm were also differentially expressed. Genes related to aquaporins, expansins, pectinesterases and endoglucanase were up-regulated, which would promote cell elongation. These results lay a foundation for further dissection of the regulatory mechanisms underlying shoot elongation at elevated temperatures.

## Introduction

Plants are able to adjust their growth to high temperature^1–4^. A set of specific responses, including hypocotyl elongation, petiole elongation, and hyponastic growth and result in a plant with an altered morphology, occur when plants are grown under different ambient temperatures^5^. Elongation of hypocotyl is an important effect in response to high ambient temperature in *Arabidopsis thaliana*^6^. It might be considered that elongated hypocotyl elevates the sensitive meristematic and photosynthetically active tissues away from the heat-adsorbing soil and may allow the plant to take better advantage of the cooling effect of moving air^4^. Phytochrome B (phyB), as a thermosensor, directly regulated the transcription of key target genes in a temperature dependent manner^7^. The bHLH transcriptional regulator PIF4 (Phytochrome interacting factor 4), regulated by phyB, is a pivotal regulator of plant growth. Present studies indicated the phyB-PIF4-auxin (phytochrome B- Phytochrome interacting factor 4) module is as a complex network that governs plant growth response to temperature^5,6^.

Present studies have revealed that various heat stress response factors (HSFs) and heat-shock proteins (HSPs) take part in ambient temperature-regulated growth^8–10^. HSP90 interacted with the auxin co-receptor F-box protein TIR1 at higher ambient temperature, to regulate plant growth^8^. Interestingly, it has been reported that HSP90 interact with BIN2, a regulator of brassinosteroid (BR) signaling, to regulate the BR signaling^11^. Large-scale changes to transcriptome in response to elevated temperature were triggered by HSF1 in *Arabidopsis thaliana*, by causing a rapid and dynamic eviction of H2A.Z (histone 2A.Z) nucleosomes at target genes in the light^12^. The thermosensor function of phyB is dependent on dark reversion^7^, while plants experience higher temperatures in sunlight. It is likely an additional temperature-sensing mechanism during the day, such as the antagonistic effects of H2A.Z and HSF1 in response to temperature^12^.

Circadian clock-related genes also play an important role in plant growth in response to elevated temperature^13–16^. The multipurpose signaling module DET1-COP1-HY5 (De etiolated 1-constitutive photomorphogenic 1-elongated hypocotyl 5) modulates PIF4 in response to light and temperature signaling^17^. DET1/COP1 stabilized the PIF4 protein at higher temperature, promoting temperature-responsive growth.

Plant hormones also involved in plant growth and development^18^. Genes related to hormone biosynthesis and signaling are the predominant targets of PIF4 during temperature-induced elongation growth^19^. Various genes involved in auxin biosynthesis or signaling participate in elongation growth response to elevated temperature, such as YUCCA8, TAA1 (tryptophan aminotransferase of Arabidopsis 1), AUX/IAA (auxin/indole-3-acetic acid), ARF (auxin response factor) and SAUR (small auxin up RNA)^20,21^. GA (gibberellin) and BR also affect temperature-induced growth^19,22,23^.

The elongated growth is regulated by either making more cells or inducing the enlargement of these existing cells. Aquaporins play pivotal roles in plant growth and development, especially in elongation growth. Aquaporin promoted hypocotyl cell elongation through increasing cell turgor^24,25^. Moreover, the activity of aquaporin was regulated by hormonal signals, temperature or light^26^. *AtTIP5;1* null mutation resulted in a dwarf phenotype with short cell in hypocotyls^25^. Additionally, the expression of *AtTIP5;1* was up regulated through application of GA_3_^25^. The rate of plant cell growth was controlled by cell wall extensibility. Expansin promoted cell elongation by altering cell wall extensibility^27–29^. In addition, genes encoding polygalacturonase (PG), pectinesterase (PE) or endoglucanase (EG) were also involved in cell elongation, that required cell wall weakening^30–32^.

A temperature-sensitive mutant in peach, SD9238 was found by the Zhengzhou Fruit Research Institute, Chinese Academy of Agricultural Sciences^33^. Genetic analysis indicated that this trait was controlled by a single dominant gene. Further substantiation for temperature sensitive of the mutant was obtained from temperature experiments (23, 26, 28, 30 and 33 °C)^33^. The extremely shortened internodes occurred when the temperature is below 30 °C. This creates an urgent need to elucidate the molecular mechanisms of shoot elongation response to high temperature. In this study, we presumed that the performance the shoot development of ‘Zhongyoutao 14’, which was derived from SD9238 and possess the temperature-sensitive characteristic, and transcript profiles of shoot at different period of ‘Zhongyoutao 14’ were analyzed under filed condition. The alteration of internode length in response to warm temperatures provides an opportunity to both understand the mechanisms regulating shoot elongation and growth responses to temperature.

## Results

### Terminal internode length was temperature dependent and determined by cell length

**Figure 1:**
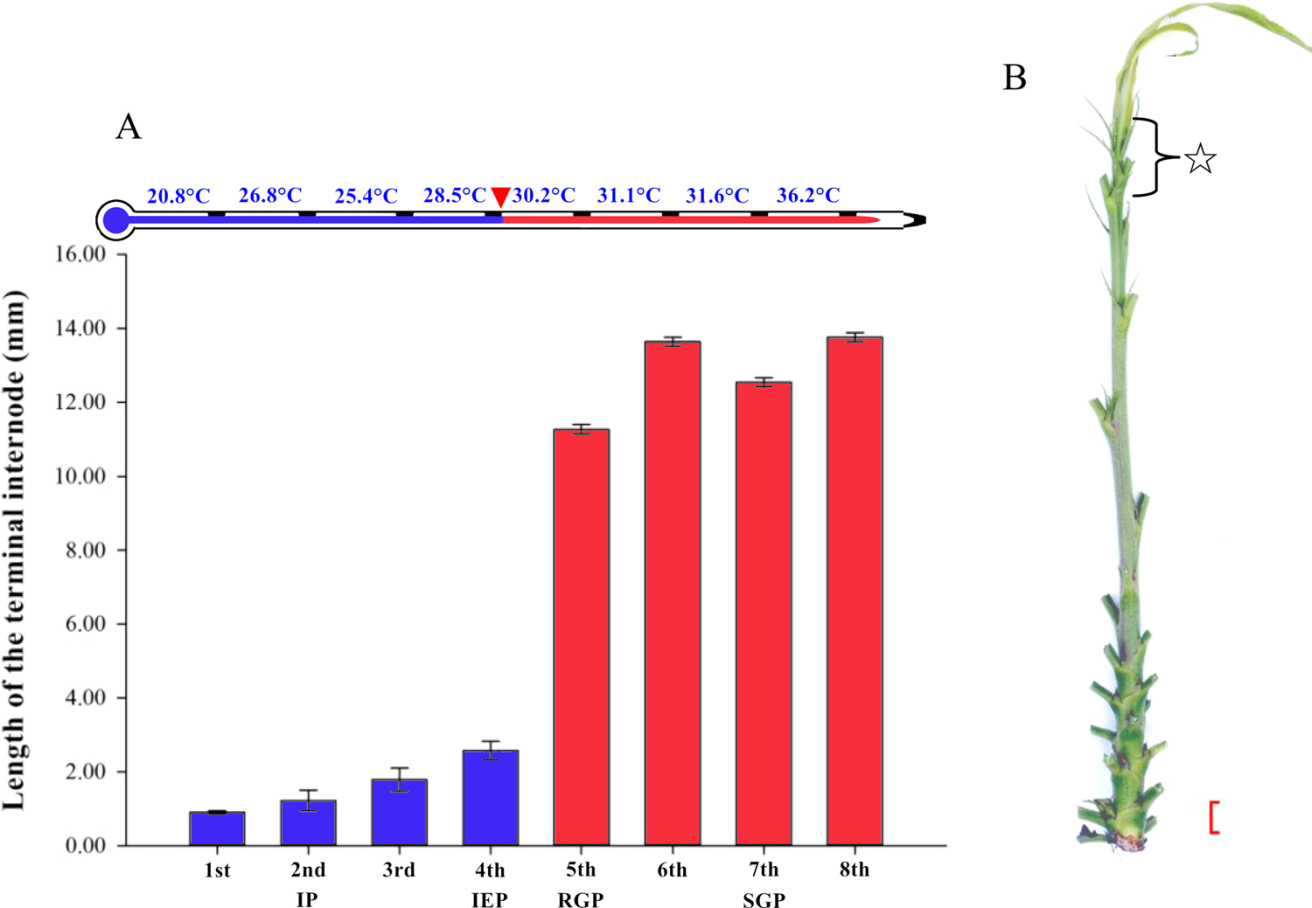
Phenotypic characterization of temperature-sensitive peach shoot. (**A**) Representative images of the terminal internodes at different stages (from 1st to 8th) aligned with the corresponding measurements of their average length (n = 6). (**B**) Image of a shoot at 9th. IP: initial period; IEP: initial elongation period; RGP: rapid growth period; SGP: stable growth period. The temperature showed the average maximum temperature of previous week (AMTPW). ^☆^Terminal internode; Bar = 5 mm.

The length of the terminal internode was measured from 15 Apr to 3 Jun per seven-day period (Fig. 1A). Meanwhile, the maximum ambient temperature in the orchard was recorded daily during this period (Table S1). During the first four period, the terminal internodes length (TIL) was between 0.90 mm and 2.57 mm. Especially the TIL was not obviously different between the second period (initial period, IP) and the third period. While, the terminal internode became slightly longer in the fourth period (initial elongation period, IEP). It’s worth noting that the TIL was significantly greater in the fifth period (rapid elongation period, RGP). The internodes could be divided into two groups based on the length (Fig. 1B). At bottom of a shoot, there were a few internodes that remained shorter. After those, the length of internodes was longer. The average maximum temperature of previous week (AMTPW) began to be higher than 30 °C in the first day of RGP (Table S1). During the later three periods, the TIL was first increased in the sixth period than RGP and then barely changed in seventh (stable growth period, SGP) and eighth periods. Base on above analysis, it’s easy to find that 30 °C is a key temperature to promote rapid growth of terminal internode in ‘Zhongyoutao 14’. And it speculated the growth of terminal internode might be as a result of temperature changes during the different periods (former lower and later higher).

Subsequently, the correlation analysis of TIL and temperature was conducted. The results indicated that there was a significantly positive correlation between the TIL and AMTPW and the maximum temperature beginning three days prior to the measuring day (DPM) (Table 1). This correlation suggested that the elongation of terminal internode might be regulated by temperature and that rapid growth might be induced by the maximum temperature (above 30 °C).

**Table 1:** Correlation coefficients between terminal internode length and daily maximum temperature.

	Previous Weekly Maximum Temperature	Daily Maximum Temperature			
		4 DPM	3 DPM	2 DPM	1 DPM
Terminal internode length	0.850**	0.632	0.900**	0.971**	0.850**

N = 8; **significant difference at P < 0.01; DPM: day prior to the measuring day. The correlation analyses were determined by Pearson correlations.

**Figure 2:**
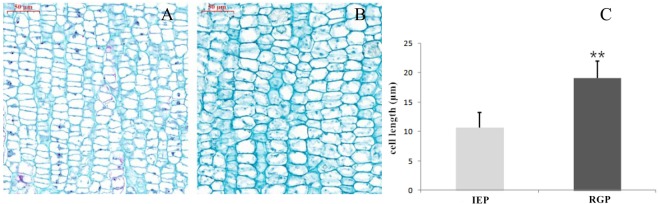
The cell length in shoot tips at IEP and RGP on ‘Zhongyoutao 14’ peach. Paraffin-sectioned images at (**A**) IEP and (**B**) RGP were used to measure the average length of the parenchyma cells (**C**) at each stage. The values represent the average of ten biological replicates (n = 10) and error bars represent the standard deviation. **Indicates significant difference at P < 0.001 between the two stages as determined by Student’s test.

At IEP and RGP, about 5 mm of the tip of the terminal internode was used to make paraffin sections for measuring the length of cells in the shoot tip (Fig. 2). At IEP, the average parenchyma cell length within the first internode was significantly shorter (P < 0.001) than cell length of the new internode one week later (RGP). This indicated that the reduced cell length is the main reason underling the shorter TIL before IEP.

### Differentially expressed genes (DEGs) involved in shoot elongation of temperature-sensitive peach

In this study, 12 cDNA libraries were sequenced from peach shoot tips harvested from four key periods, including IP, IEP, RGP and SGP. On average, about 6.64 Gb clean bases were generated for each library (Table S2). For each replicate, about 88.27% of the clean reads were successfully mapped to the peach genome (Prunus_persica_v2.0). By transcriptome analysis, the DEGs between different stages were identified. In the three pairwise comparisons, there were 1942 DEGs between IP and IEP, 1475 DEGs between IEP and RGP, and 100 DEGs between SGP and RGP (Fig. S1). To confirm the reliability of the RNA-seq data, the expression levels of 15 genes related to the following analysis were examined using RT-qPCR. The expression patterns of these genes were consistent in the RT-qPCR and RNA-seq datasets (Fig. S2), indicating that the analysis of DEGs using the RNA-seq data was reliable. The number of DEGs in the shoot tips decreased with time. The differences in transcript levels occurred much earlier than differences in phenotype. Since elongation of the terminal internode is the result of response to higher temperature (Table 1), the following analysis focused on DEGs related to temperature perception, signaling and elongation growth according to their function as annotated.

### DEGs related to heat shock proteins (HSPs) and heat shock transcription factors (HSFs) during shoot elongation in temperature-sensitive peach

**Figure 3:**
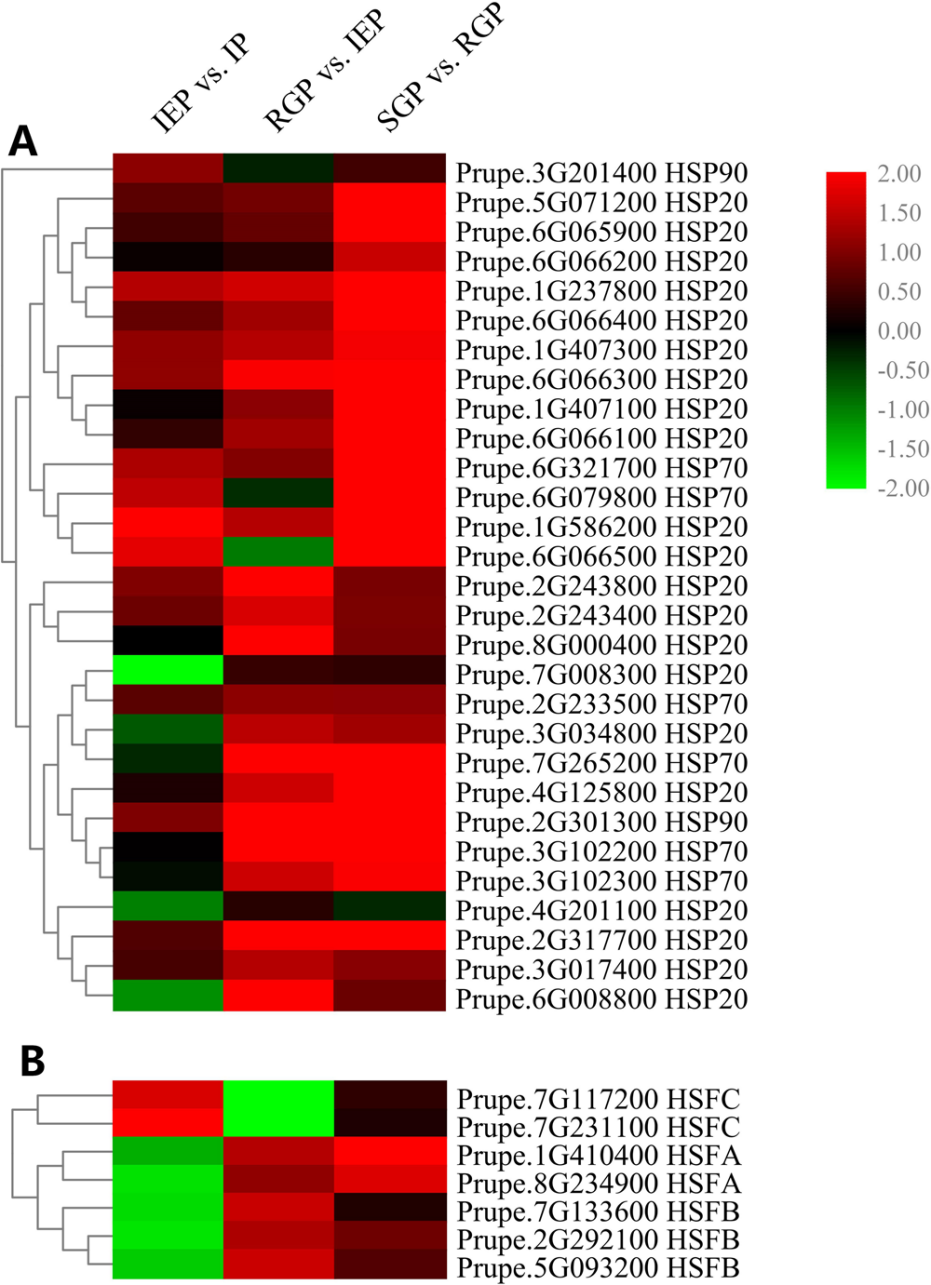
Heat maps of the DEGs encoding HSPs and HSFs during shoot elongation in temperature-sensitive peach. (**A**) Heatmap of the DEGs encoding HSPs (heat shock proteins). (**B**) Heatmap of the DEGs encoding HSFs (heat shock factors). Red and green colors indicate up- and down- regulated transcripts, respectively, from the three comparisons (log2-fold change).

In total, 29 HSP-related DEGs were identified across the different stages, including two HSP90s, six HSP70s and 21 HSP20s (Fig. 3A). Most of the gene expression levels showed a progressive increase, especially after RGP. Between IEP and SGP, the majority of genes were sharply increased, such as two HSP20s, Prupe.2G243800 (9.50-fold increase) and Prupe.8G000400 (10.86-fold) and one HSP70, Prupe.7G265200 (11.56-fold).

The HSPs are transcriptionally induced by HSF upon activation by heat stress or ambient temperature. Seven HSFs were detected among the DEGs. The HSFA and HSFB groups were consistently up-regulated with time, while the HSFC group was down-regulated between IEP and RGP (Fig. 3B). Among these genes, the HSFAs Prupe.1G410400 and Prupe.8G234900 were up-regulated by 2.69-fold and 2.19-fold, respectively, between IEP and RGP.

### DEGs involved in plant hormone signal transduction and biosynthesis during shoot elongation in temperature-sensitive peach

**Figure 4:**
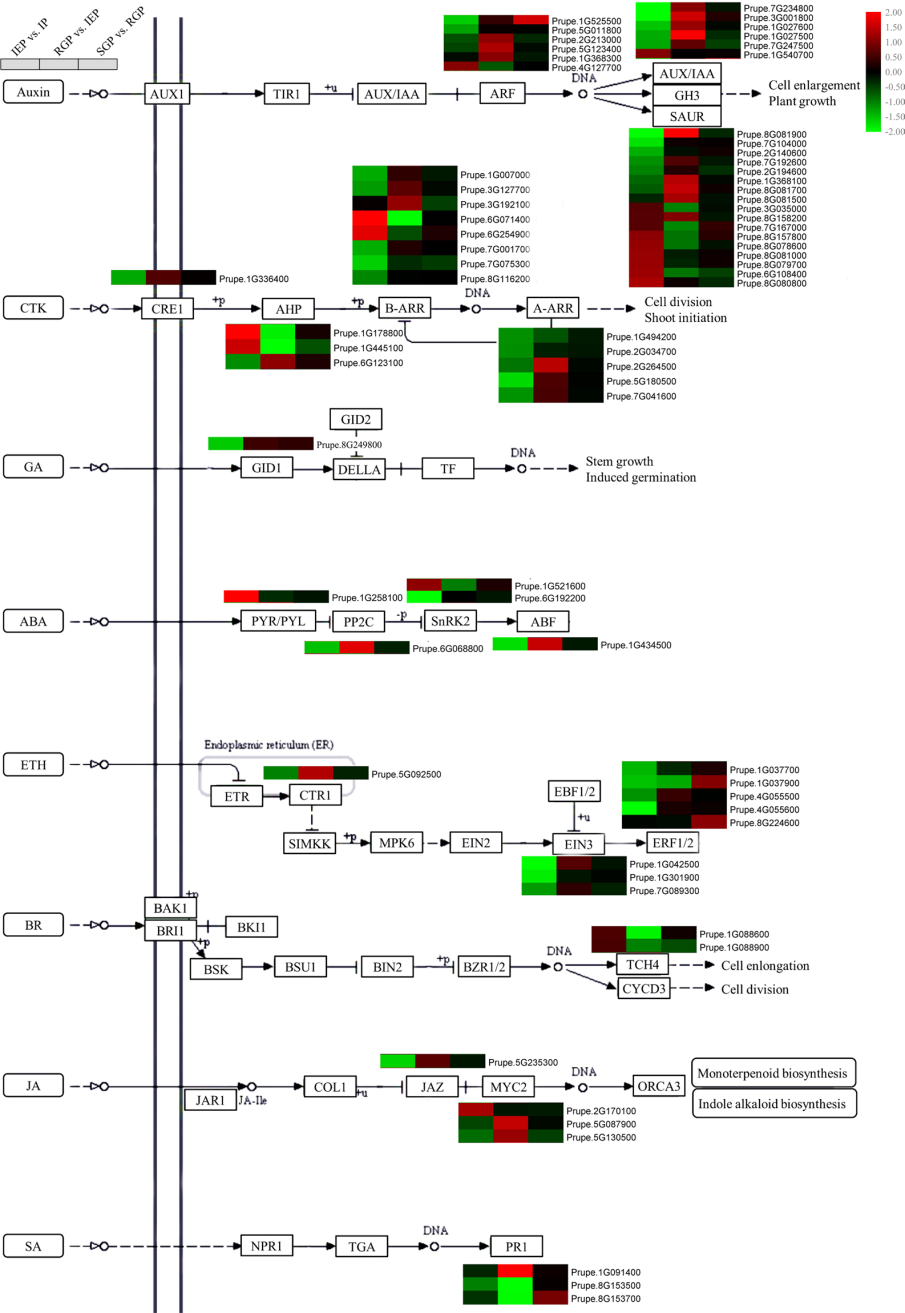
Heat maps of DEGs involved in plant hormone signal transduction during shoot elongation in temperature-sensitive peach. Red and green colors indicate up- and down- regulated transcripts, respectively, from the three comparisons (log2-fold change). CTK: cytokinine, GA: gibberellin, ABA: abscisic acid, ET: ethylene, BR: brassinosteroid, JA: jasmonic acid, SA: salicylic acid. ARF: auxin response factor (Prupe.4G127700; Prupe.5G011800; Prupe.1G525500; Prupe.1G368300; Prupe.2G213000; Prupe.5G123400). AUX1/IAA: auxin-responsive protein IAA (Prupe.1G540700; Prupe.7G247500; Prupe.1G027500; Prupe.1G027600; Prupe.3G001800; Prupe.7G234800). SAUR: SAUR family protein (Prupe.7G167000; Prupe.3G035000; Prupe.6G108400; Prupe.8G078600; Prupe.8G157800; Prupe.8G079700; Prupe.8G081000; Prupe.2G140600; Prupe.7G104000; Prupe.8G080800; Prupe.2G194600; Prupe.7G192600; Prupe.1G368100; Prupe.8G081500; Prupe.8G081700; Prupe.8G081900; Prupe.8G158200). CRE1: cytokinin receptor (Prupe.1G336400). AHP: histidine-containing phosphotransfer peotein (Prupe.1G445100; Prupe.1G178800; Prupe.6G123100). A-ARR: two-component response regulator ARR-A family (Prupe.1G494200; Prupe.2G034700; Prupe.1G261500; Prupe.7G041600; Prupe.5G180500; Prupe.2G264500). B-ARR: two-component response regulator ARR-b family (Prupe.6G071400; Prupe.6G254900; Prupe.7G075300; Prupe.8G116200; Prupe.7G001700; Prupe.1G007000; Prupe.3G127700; Prupe.3G192100). GID1: gibberellin receptor GID1 (Prupe.8G249800). PYR/PYL: abscisic acid receptor PYR/PYL family (Prupe.1G258100). PP2C: protein phosphatase 2C (Prupe.6G068800). SnRK2: serine/threonine-protein kinase SRK2 (Prupe.1G521600; Prupe.6G192200). ABF: ABA responsive element binding factor (Prupe.1G434500). CTR1: serine/threonine-protein kinase CTR1 (Prupe.5G092500). EIN3: ethylene-insensitive protein 3 (Prupe.1G042500; Prupe.1G301900; Prupe.7G089300). ERF: ethylene responsive transcription factor (Prupe.1G037900; Prupe.1G037700; Prupe.8G224600; Prupe.4G055600; Prupe.4G055500). TCH4: xyloglucan:xyloglucosyl transferase TCH4 (Prupe.1G088600; Prupe.1G088900). JAZ: jasmonate ZIM domain-containing protein (Prupe.5G235300). MYC2: (Prupe.2G170100; Prupe.5G087900; Prupe.5G130500). PR1: pathogenesis-related protein 1 (Prupe.8G153500; Prupe.8G153700; Prupe.1G091400).

Numerous DEGs involved in phytohormone signal transduction and biosynthesis were identified. Genes in these pathways showed significant dynamic changes during shoot development (Fig. 4 and Table S3). The DEGs were involved in auxin, CTK (cytokinin), GA, BR, ETH (ethylene), JA (jasmonic acid) and SA (salicylic acid) biosynthesis and signaling.

Twenty-nine DEGs related to auxin signaling were observed. The majority of them were up-regulated at RGP, with particularly high levels in an AUX1/IAA gene (Prupe.1G027500) and a SAUR gene (Prupe.8G081900), both of which had more than a 3.5-fold change. The TAA1 (Prupe.8G041200) was highly expressed at IEP, but down-regulated at RGP.

Three of the four identified IPTs (adenosine-phosphate isopentenyl-transferase), which encode a key enzyme in CTK biosynthesis, were down-regulated by 1.91- to 3.73-fold from IEP to RGP, with the exception being Prupe.1G151100. The expression of CKX (cytokinin oxidase/dehydrogenase, Prupe.7G208400) was up-regulated at this stage. Of nine CTK glycosylation genes, six genes showed up-regulation, while the other three showed down-regulation. Six CTK signaling genes showed significantly differential expression between IEP and RGP, with a particularly large changes of expression in an AHP (histidine-containing phosphotransfer, Prupe.1G445100) and a B-ARR (ARR-B-type two-component response regulator, Prupe.6G071400), both showing a more than 8-fold degree of down-regulation. This suggested that CTK biosynthesis and signaling might have been suppressed between IEP and RGP.

In the GA biosynthesis and signaling pathways, the genes were differentially expressed between IP and IEP. The biosynthetic genes GA20ox (GA 20-oxidase, Prupe.1G442200) was up-regulated, but GA3ox (GA 3-oxidase, Prupe.3G075600) was down-regulated. Meanwhile, GA2ox (GA 2-oxidase, Prupe.4G150200), which deactivates the bioactive GA, was down-regulated at the same time. This indicated that the balance between GA biosynthesis and catabolism was intimately controlled during temperature-sensitive shoot elongation. No GA signaling genes were found to be differentially expressed between IEP and RGP.

Genes involved in BR biosynthesis were down-regulated during temperature- sensitive shoot development, including one DWF4 (dwarf4), one CPD (constitutive photomorphogenesis and dwarfism), three ROT3 (rotundifolia 3), and one BR6ox (brassinosteroid-6-oxidase) genes. Prupe.7G153500 (DWF4) was significantly down- regulated, by 2.19-fold. Prupe.1G291100 and Prupe.1G296600, two BAS1 (PHYB activation tagged suppressor 1) genes encoding BR metabolism enzymes, were also down-regulated, by 1.42- and 4.03-fold, respectively. TCH4 (two touch 4), which is regulated by BR, was also down-regulated between IEP and RGP. Down-regulation of these genes in the BR biosynthesis and signaling pathways indicated that BR biosynthesis and signaling might be suppressed during temperature-sensitive shoot elongation.

Genes related to other major phytohormones also showed large changes during temperature-sensitive shoot development. Four JMT genes (jasmonic acid carboxyl methyltransferase), which catabolize JA to form methyl jasmonate (MeJA) and serve as a critical control point for jasmonate-regulated plant responses, were up-regulated by 1.38- to 5.10-fold between IEP and RGP. Two genes encoding MYC2, which are related to JA signaling, were also up-regulated. Genes involved in ABA, ETH and SA pathways also showed down- or up-regulation during temperature-sensitive shoot development.

### DEGs involved in circadian rhythm during shoot elongation in temperature-sensitive peach

**Figure 5:**
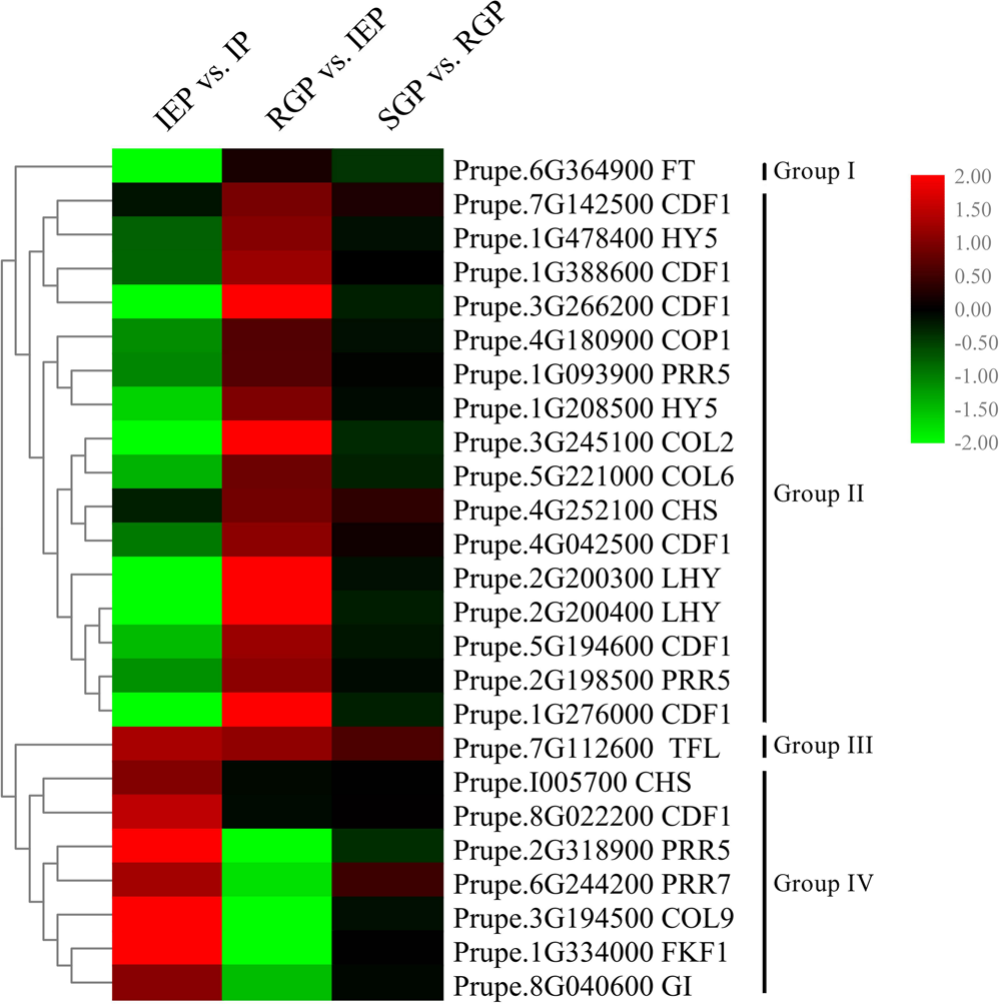
Heat maps of DEGs involved in circadian rhythm during shoot elongation in temperature-sensitive peach. Genes were classified into groups based on the transcript profile. Red and green colors indicate up- and down- regulated transcripts, respectively, from the three comparisons (log2-fold change).

The responses of plant to photoperiod and temperature are intimately associated, thus genes predicted to function in circadian responses were also sought within the transcriptome data. Twenty-five DEGs related to circadian rhythm were identified and then divided among four groups according to their expression patterns (Fig. 5). Group I contained one gene that was gradually down-regulated, namely Prupe.6G364900 (FLOWERING LOCUS T, FT). Group II contained genes exhibiting their lowest expression levels at IEP, including two LHYs, two HY5s, six CDF1 (cycling DOF factor 1), one COP1, two PRR5, one CHS (chalcone synthase) and two COLs (constans-like). Of these DEGs, the LHY-encoding genes Prupe.2G200300 and Prupe.2G200400 were up-regulated by 11.76- and 99.45-fold between IEP and RGP. The sole gene in group III, Prupe.7G112600, encoding TFL1 (TERMINAL FLOWER 1), showed a gradual increase of transcript, over the four periods analyzed. The six DEGs in group IV exhibited their highest expression levels at IEP, including PRR7 (Prupe.6G244200).

### DEGs involved in cell elongation during shoot elongation in temperature-sensitive peach

**Figure 6:**
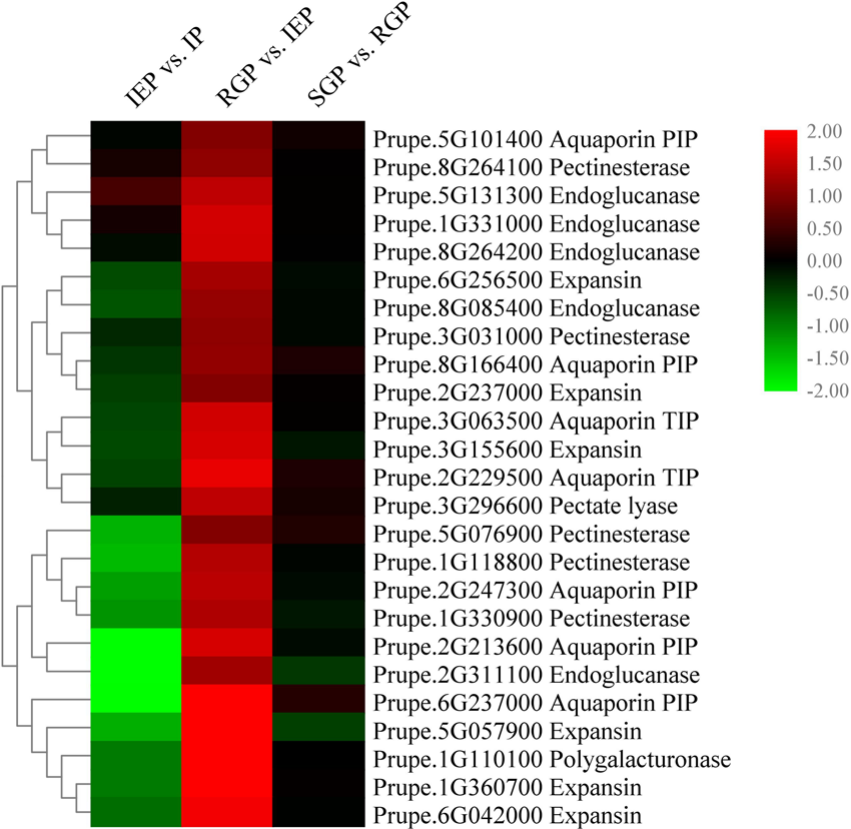
Heatmap of DEGs involved in cell elongation during shoot elongation in temperature-sensitive peach. Red and green colors indicate up- and down- regulated transcripts, respectively, from the three comparisons (log2-fold change).

In total, 25 DEGs related to cell expansion were detected during the key period (Fig. 6). Generally, all of these DEGs had similar expression patterns, with significant up-regulation between IEP and RGP. In this study, there were seven up-regulated aquaporins, including five plasma membrane intrinsic proteins (PIPs) and two tonoplast intrinsic proteins (TIPs) (Fig. 6). Prupe.6G237000 (PIP) showed the highest change of 5.44-fold. Expansins are regarded as cell wall loosening proteins that can weaken noncovalent bonds between the cell wall matrix polymers to extend the growing cell. Six homologs of expansin were up-regulated, by 2.03- to 4.22-fold.

There were 12 DEGs related to cell wall synthesis, modification or hydrolysis, including one polygalacturonase (PG), one pectate lyase (PL), five Pectinesterases (PE) and five endoglucanases (EG) (Fig. 7). Their transcript levels at RGP were at least 2.03-fold higher compared to at IEP, with Prupe.1G110100 (PG) showing the highest fold change (5.82-fold).

## Discussion

Environmental factors are important signals regulating shoot elongation, especially light and temperature. Compared with the comparatively well-defined light signaling pathways, molecular characterization of temperature perception and signaling is harder to resolve^34^. Although several studies of ambient-temperature perception and signaling have been reported^3,17,19,20,22,35,36^, the fundamental mechanisms behind morphological changes remain unclear. SD9238 as a mutant that remained small in stature, was discovered in seedling population in 1993. ‘Zhongyoutao 14’ was derived from a cross between 90-1-25 and SD9238^37^. Further characterization showed that the temperature-sensitive peach exhibited extremely shortened internodes at lower temperatures (below 30 °C), but longer internodes at temperature above 30 °C^33^. In this study, we observed that the length of the shoot in ‘Zhongyoutao 14’ sharply increased from IEP to RGP. It was also observed that the AMTPW was over 30 °C at IEP (Fig. 1; Table S1). These indicated that the increase in temperature might be a factor inducing rapid growth and that 30 °C might be a key temperature. In *Arabidopsis thaliana*, the warm temperature associated with the morphology change mainly ranges below this temperature^6^.

Transcriptome profiling was carried out to understand the molecular mechanisms behind the shoot elongation in temperature-sensitive peach. It is noteworthy that a greater number of DEGs were detected before RGP, while fewer DEGs were observed between RGP and SGP. This indicated that a number of genes were involved in shoot elongation in peach and that transcriptional regulation may play a vital role in temperature-induced shoot elongation of peach. Genes involved in temperature sensing or signaling, influenced the expression of ambient temperature-induced genes, such as *PIF4, HSF1*, triggering large-scale changes to the transcriptome^1,12^. The signaling or biosynthesis of hormones including auxin, BRs and GAs were impinged by ambient temperature, which modulated the growth rate^19^. Circadian clock cue was always integrated with temperature to regulate plant growth, for example flowering and internode elongation^36,38,39^. Taken together, DEGs involved in these pathways might participate in the shoot elongation of temperature-sensitive peach.

PIF4 and phyB were identified as hub genes that play extremely important roles in temperature-induced elongation, especially under diurnal temperature fluctuation^7^. Interestingly, their expression levels did not significantly change in temperature-sensitive peach shoot tips when higher ambient temperatures induced longer terminal internodes (data not shown) in this study. Furthermore, the elongation of the temperature-sensitive peach internode was promoted by the maximum temperature in the days leading up to measurable changes. These results indicated that there is likely a different mechanism that sense temperature change in this peach line.

In *Arabidopsis thaliana*, the expression patterns of HSFs, such as HSFA2, HSFA7a, HSFB1 and HSFB2b, are induced by warm temperature^40^. In addition, HSFB1 and HSFB2b, as transcriptional repressors, act in a negative feedback loop to these genes^40^. In our study, the expression of HSFAs and HSFBs were up-regulated in shoots after seasonal temperatures began to rise, which indicated that the elevated temperature induced the expression of HSFs in temperature-sensitive peach. The expression of HSFCs showed the opposite expression pattern to HSFAs and HSFBs, however, the function of HSFCs in temperature-induced elongation is less understood. In the HSFA1 quadruple knockout mutants of *Arabidopsis thaliana*, the morphologies were altered and the growth rates were decreased, and transcriptomics analysis showed that over 65% of the heat stress-related genes were dependent of HSFA1^41^. HSFA1 is activated by the temperature in the light, triggering a large-scale change in the transcriptome by removing the H2A.Z nucleosome on genes^12^. However, the expression levels of H2A.Z were not significantly changed in our study, perhaps because the H2A.Z response to temperature is independent of transcription^1^. Therefore, it was speculated that HSFAs might trigger changes in the transcriptome in response to elevated temperature.

In this study, the expression patterns of most genes encoding HSPs increased gradually with the increase of ambient temperature. Six differentially expressed HSP70s were highly homologous with At3g12580 (e-value = 0.0). Kumar *et al*. has revealed that with increasing ambient temperature, At3g12580 (HSP70) was strongly induced, but the other members of the HSP70 family were not^1^. This suggested that in peach there might be more than one HSP70 within the ambient temperature-sensing pathway. HSP90 (Prupe.2G301300) was also induced in peach in response to warmer ambient temperature. HSP90s, as molecular chaperones, not only play central roles in heat sensing and signaling, but also participate in auxin and BR signaling^8,11,42^. In *Arabidopsis thaliana*, the accumulation of the TIR1 auxin co-receptor was dependent on HSP90 at higher temperature^8^. This suggested that the warm temperature-inducible HSP90 might enhance auxin signaling. In peach, Prupe.8G000400, encoding HSP20, showed the highest fold-change in expression between IEP and RGP. It seems that HSPs could provide crosstalk between temperature sensing and hormone signaling during the regulation of shoot elongation.

Genes related to eight phytohormone pathways showed up- or down-regulated during shoot elongation in temperature-sensitive peach. Previous studies showed that ambient temperature regulates growth by cell elongation through impacting the auxin, GA and BR pathways^19^. In our study, DEGs related to auxin signaling, such as ARF, AUX/IAA and SAUR, were significantly up-regulated. These changes suggested that auxin signaling was enhanced, which would then induce rapid growth of temperature-sensitive peach.

Interestingly, genes in the BR biosynthesis and signaling pathways were down-regulated during temperature-sensitive peach development, such as DWF4, BR6OX1 and TCH4. In *Arabidopsis thaliana*, it has been shown that the biosynthesis of BR was increased at elevated temperature^19^. The role of BR in shoot elongation of temperature-sensitive peach requires further study.

One DEG associated with the GA biosynthesis pathway, GA2ox, was up-regulated from IP to IEP. Between IEP and RGP, the expression of GA pathway genes showed no drastic changes. It has been found that GA biosynthesis was induced by high temperature^19^.

DEGs involved in the CTK biosynthesis and signaling pathways were down-regulated. The expression patterns of genes involved in ETH, JA and SA pathways, were also affected. This suggested that hormone signals interact at multiple levels during temperature-induced changes in TIL. The auxin response and CTK biosynthesis pathway are regulated by opposing feedback mechanisms that maintain appropriate auxin and CTK concentrations in developing shoot tissues in *Arabidopsis*^43^. Attenuated CTK biosynthesis and signaling might be induced by increased auxin signaling in our study. This is consistent with the shoot elongation being caused by cell elongation rather than cell division. The above results further suggested that the hormone pathways interact with each other and together mediate the shoot elongation in temperature-sensitive peach.

Genes related to circadian rhythm play an important role in temperature sensing and temperature-induced elongation^35,36,44^. Recent studies have suggested that there is tight coordination between light signaling and temperature responses^15,17,45,46^. In this study, there were several DEGs involved in the circadian rhythm pathway, such as LHY, HY5, PRR7, FKF1 and GI. Two of these DEGs, Prupe.2G200300 and Prupe.2G200400, were annotated as LHY. In *Arabidopsis thaliana*, LHY had the opposite expression pattern (down-regulated) as the temperature increases to maintain robust rhythms^47^. The up-regulation of LHYs in our study might be due to decreased expression of PRR7. AtPRR7 was shown to negatively regulate AtLHY in response to ambient temperature^44^. Transcript levels of *AtHY5* was also inhibited by elevated temperature^48^, which then relieved the repression on PIF4 target genes by HY5, in order to regulate temperature-induced elongation growth^35^. In ‘Zhongyoutao 14’, the expression levels of two HY5s were up-regulated with the temperature increasing, which suggested that the HY5s’ regulating mechanism in this temperature sense mutant might be different. The above results indicated that the different expression of genes related to circadian rhythm might be a response to ambient temperature in temperature-sensitive peach.

FT promotes the transfer from vegetative to reproductive growth, whereas TFL1 represses this transition^49^. The expression patterns of FT and TFL1 were opposite in our study. The FT/TFL1 gene family plays diverse roles in multiple developmental processes other than flowering regulation^50^. In *Populus*, TFL1 might promote stem elongation^51^. Up-regulation of TFL1 in our study implied that TFL1 might promote temperature-sensitive peach shoot elongation.

In peach, a brachytic GA-insensitive dwarf, was caused by shorter cell length^52^. In this temperature-sensitive peach, the shorter TIL phenotype under lower temperature was also caused by shorter cells in the shoot tip at early stages.

Seven homologies of aquaporin, including five PIPs and two TIPs, were significantly up-regulated during temperature-sensitive shoot elongation. Prupe.6G237000 (a PIP) showed the highest fold change (5.44-fold) between IEP and RGP. Ludevid *et al*. found that *γ-TIP* (*AtTIP1;1*) was primarily expressed during cell enlargement^53^. In *Arabidopsis*, lines overexpressing *AtTIP5;1* exhibited significantly longer hypocotyl than WT plants^25^. Muto *et al*. found that the expression patterns of *OsTIP1;1*, *OsTIP2;2*, *OsPIP1;1*, *OsPIP2;1* and *OsPIP2;2* were associated with stem elongation^24^.

Expansins, pectinesterases and endoglucanases, cell wall proteins or enzymes involved in cell-wall loosening^54^, were up-regulated at RGP. Prupe.1G110100 (PG) increased 5.82-fold between IEP and RGP. In *Oryza. sativa*, longer coleoptile and mesocotyl were observed in lines overexpressing *OsEXP4*, with corresponding larger cell size^27^. The above results further suggested that cell size was the cause of a semi-dwarf phenotype, which might be regulated by aquaporins, expansins, pectinesterases and endoglucanases.

## Conclusion

In this study, the phenotype changes of temperature-sensitive peach were due to response to elevated ambient temperature. The internodes were divided into two groups according to the length. Lower temperature (below 30 °C) in former periods resulted in shortened internodes, while higher temperature (over 30 °C) in later periods significantly promoted the terminal internode elongation. The DEGs related to temperature perception and signaling, plant hormone signal transduction and biosynthesis, circadian rhythm, and cell wall, were explored as relevant to temperature-induced elongation. Several genes such as HSFs, HSPs, AUX/IAAs, LHYs, TFL1, EXP and aquaporins were up-regulated. There might be another thread of temperature sensing differed from phyB-PIF4-auxin module, due to no significantly changes in expression levels of phyB and PIF4. Characterization of these cellular and transcriptomic changes provides a valuable resource for further dissection of the molecular mechanisms controlling shoot elongation at elevated temperatures in temperature-sensitive peach.

## Materials and Methods

### Plant materials

The temperature-sensitive peach cultivar ‘Zhongyoutao 14’ (*Prunus persica*, 90-1-25 × SD9238)^37^, was grown at the Experimental Station of the Horticulture College, Henan Agricultural University (Zhengzhou, China). TIL was successive measured eight times from 15 Apr to 3 Jun in seven-day period using a vernier caliper. The terminal internode initiated at the measuring day was marked with a tag, the length of which was continuously measured for three weeks. Six shoots per tree at different positions were measured. Temperature data were collected by an RR9310 thermometer (Rainroot Ltd., China).

### RNA sampling

For RNA extraction, twenty shoot tips were collected from different points around each tree at 10 AM and combined. Samples were collected at four periods, including initial period (IP, 2nd), initial elongation period (IEP, 4th), rapid growth period (RGP, 5th) and stable growth period (SGP, 7th). Three trees were combined for one replicate and three biological replicates were taken, for a total of nine trees sampled at each stage. All samples were immediately frozen in liquid nitrogen and stored at −80 °C until use.

### Paraffin sectioning

Shoot tips were harvested at IEP and RGP and fixed immediately in the fixative solution (formaldehyde: glacial acetic acid: 70% ethanol = 1:1:18, by volume). After 24 h, the shoot tips were dehydrated in a gradient ethanol series and further processed according to Cheng *et al*.^52^. The measurements of cell dimensions were performed by caseviewer software. The cells were selected at the same position in each section. Each cell was measured in the section and total ten cells were measured. Each value was used for the statistical analysis.

### Total RNA extraction, RNA-Seq library construction and sequencing

Total RNA was extracted from the pooled shoot tip samples harvested at IP, IEP, RGP and SGP using the Total RNA Rapid Extraction Kit (Sangon, Shanghai, China) according to the manufacturer’s instructions. Three biological replicates for each cultivar were made into 12 cDNA libraries using the RNA Library Prep Kit according to the manufacturer’s instructions (NEB, USA) and sequenced on the BGISEQ-500 Platform.

Raw reads obtained from sequencing platform were filtered through removing the reads of low-quality or containing adaptor sequence or poly-N. Clean reads were then mapped to the peach genome (https://phytozome.jgi.doe.gov/pz/portal.html#!info?alias=Org_Ppersica) using Tophat^55^. The mapped read counts, implying the gene expression level, were normalized as Fragments Per Kilobase of transcript per Million mapped reads (FPKM)^56^. Differentially expression analyses between different stages (IEP vs. IP, RGP vs. IEP, SGP vs. RGP) were performed using DEseq. 2^57^. Raw P-values were adjusted for multiple testing using a false discovery rate (FDR)^58^. Genes with a FDR of less than 0.05 and fold-changes greater than 2 were regarded as DEGs. Hierarchical clustering for DEGs was performed using TBtools^59^. The genes function was annotated by the NCBI non-redundant protein sequences (Nr), GO (Gene Ontology) and KEGG (Kyoto Encyclopedia of Genes and Genomes) database.

### Quantitative real-time RT-PCR (qRT-PCR)

One microgram of total RNA per sample was subjected to cDNA synthesis using cDNA Synthesis SuperMix (TransGen, Beijing, China) according to the manufacturer’s instructions. A SYBR green-based real-time PCR assay was carried out. The content of reaction mixtures and the amplification program were referred as described by Cheng *et al*.^52^. All analysis was repeated three times using biological replicates. The difference in the cycle threshold (Ct) between target and actin genes corresponded to the level of gene expression. A peach EF2 (elongation factor 2) gene was used as a constitutive control. All primer sequences are listed in Table S4.

## Supplementary information

Supplementary information.

Supplementary information2.

Supplementary information3.

Supplementary information4.

Supplementary information5.

Supplementary information6.
