# Supplementary figures and images for: Transcript profiling provides insights into molecular processes during shoot elongation in temperature-sensitive peach (Prunus persica)
Source: Sci Rep. 2020 May 8;10:7801. doi: 10.1038/s41598-020-63952-2 (PMC7210264; doi:10.1038/s41598-020-63952-2)

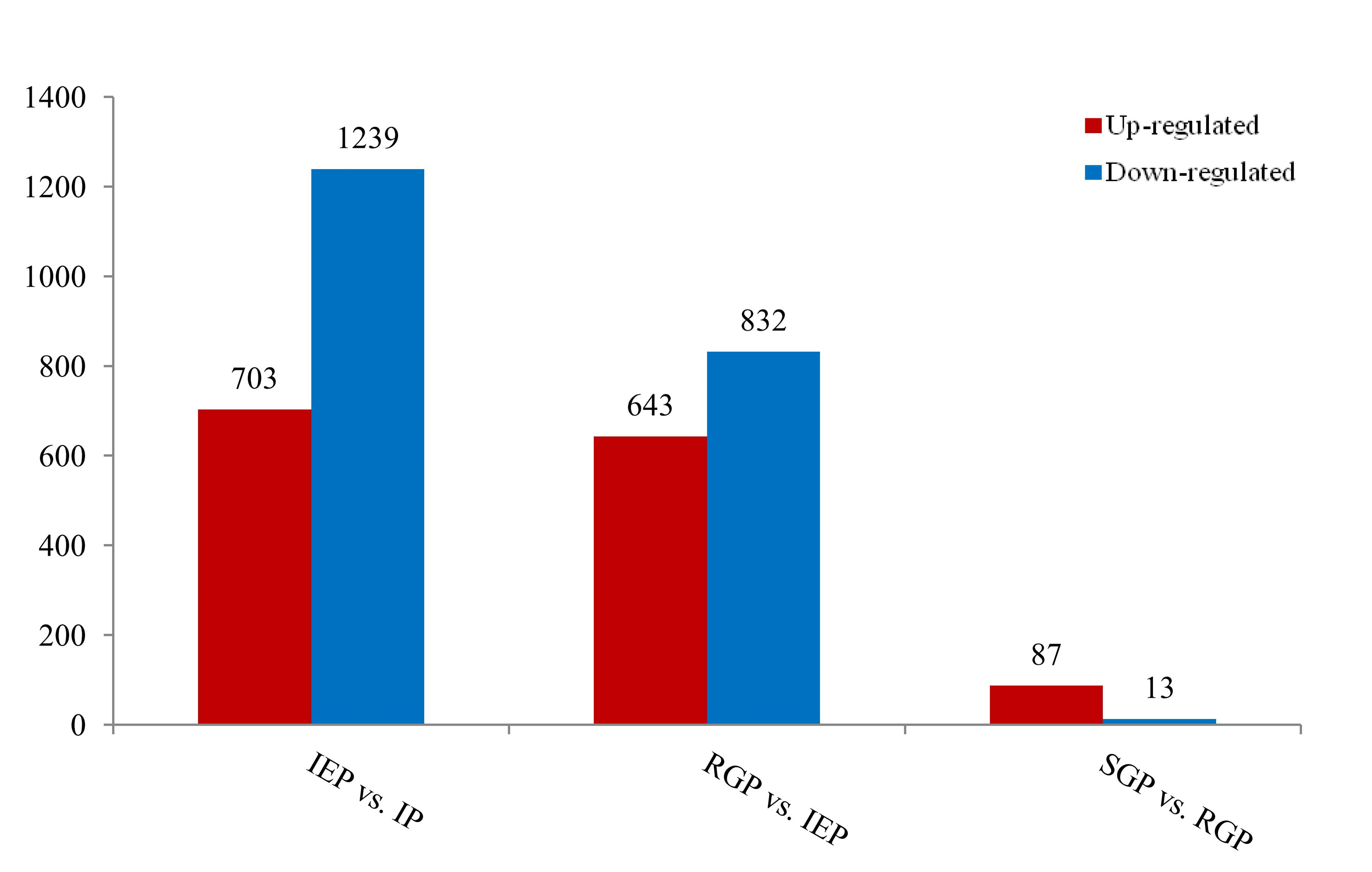

Supplement: Supplementary file 1 — Supplementary information. [file 41598_2020_63952_MOESM1_ESM.jpg]

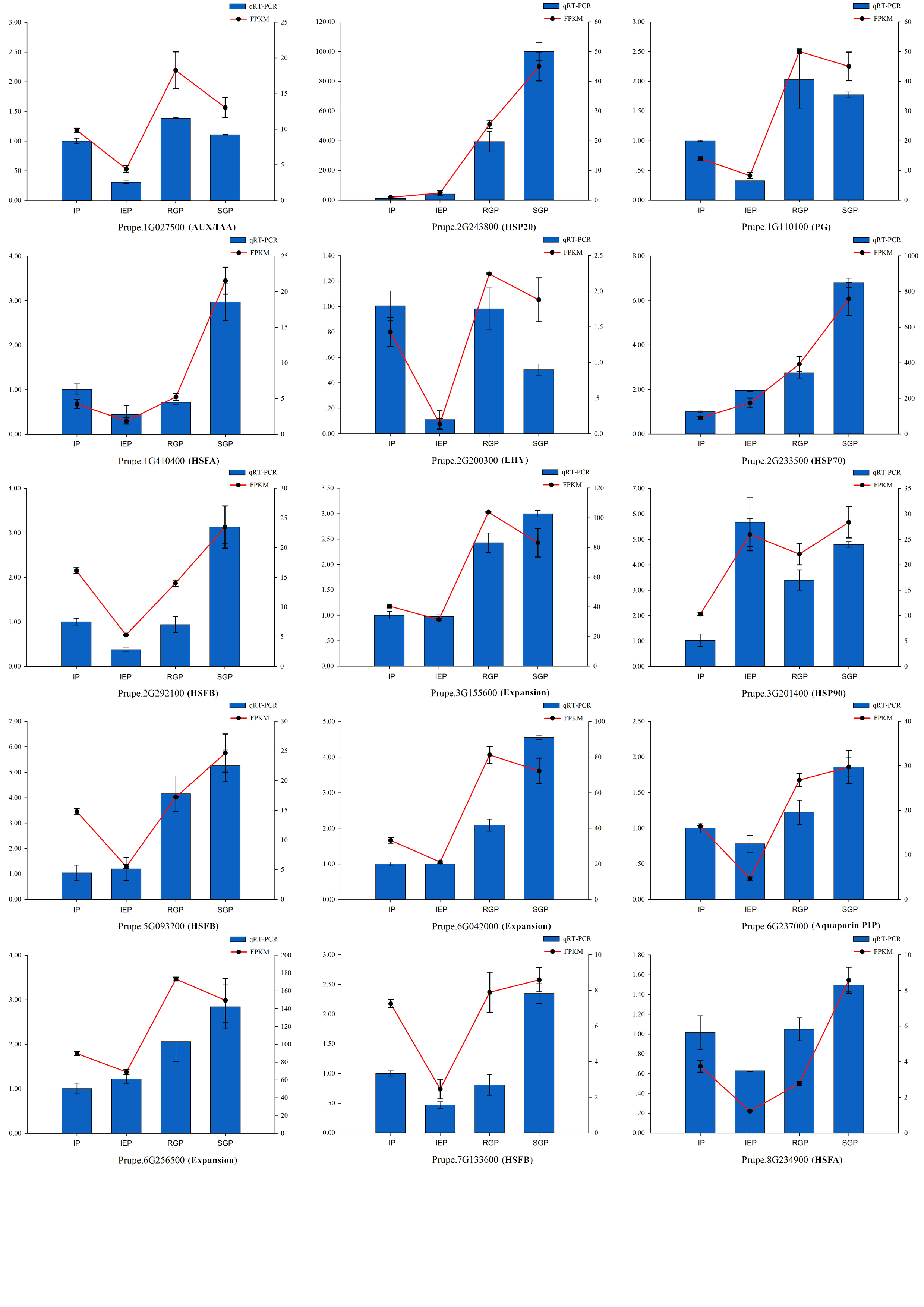

Supplement: Supplementary file 2 — Supplementary information2. [file 41598_2020_63952_MOESM2_ESM.jpg]
